# Sepsis-Induced Changes in Spectral Segregation and Kinetics of Hippocampal Oscillatory States in Rats

**DOI:** 10.1523/ENEURO.0002-23.2023

**Published:** 2023-06-19

**Authors:** Annu Kala, Susan Leemburg, Karel Jezek

**Affiliations:** Biomedical Center, Faculty of Medicine in Pilsen, Charles University, Pilsen 323 00, Czech Republic

**Keywords:** activity states, hippocampus, inflammation, oscillations, sepsis, sleep

## Abstract

Sepsis-associated encephalopathy (SAE) is a frequent severe complication of sepsis and the systemic inflammatory response syndrome, associated with high mortality and long-term neurologic consequences in surviving patients. One of the main clinical signs of SAE are discontinuous sleep periods that are fragmented by frequent awakenings. Although this brain state fragmentation strongly impacts the functionality of the nervous and other systems, its underlying network mechanisms are still poorly understood. In this work, we therefore aim to characterize the properties and dynamics of brain oscillatory states in response to SAE in an acute rat model of sepsis induced by high-dose lipopolysaccharide (LPS; 10 mg/kg). To focus on intrinsically generated brain state dynamics, we used a urethane model that spares oscillatory activity in rapid eye movement (REM)-like and nonrapid eye movement (NREM)-like sleep states. Intraperitoneal LPS injection led to a robust instability of both oscillatory states resulting in several folds more state transitions. We identified opposing shifts in low-frequency oscillations (1–9 Hz) in REM and NREM-like states under influence of LPS. This resulted in increased similarity between both states. Moreover, the state-space jitter in both states increased as well, pointing to higher within-state instability. The reduction of interstate spectral distances in 2-D state space, combined with increased within-state jitter might represent a key factor in changing the energy landscape of brain oscillatory state attractors, and hence lead to altered sleep architecture. Their emergence during sepsis might point to a mechanism underlying severe sleep fragmentation as described both in sepsis patients and SAE animal models.

## Visual Abstract

**Figure:**
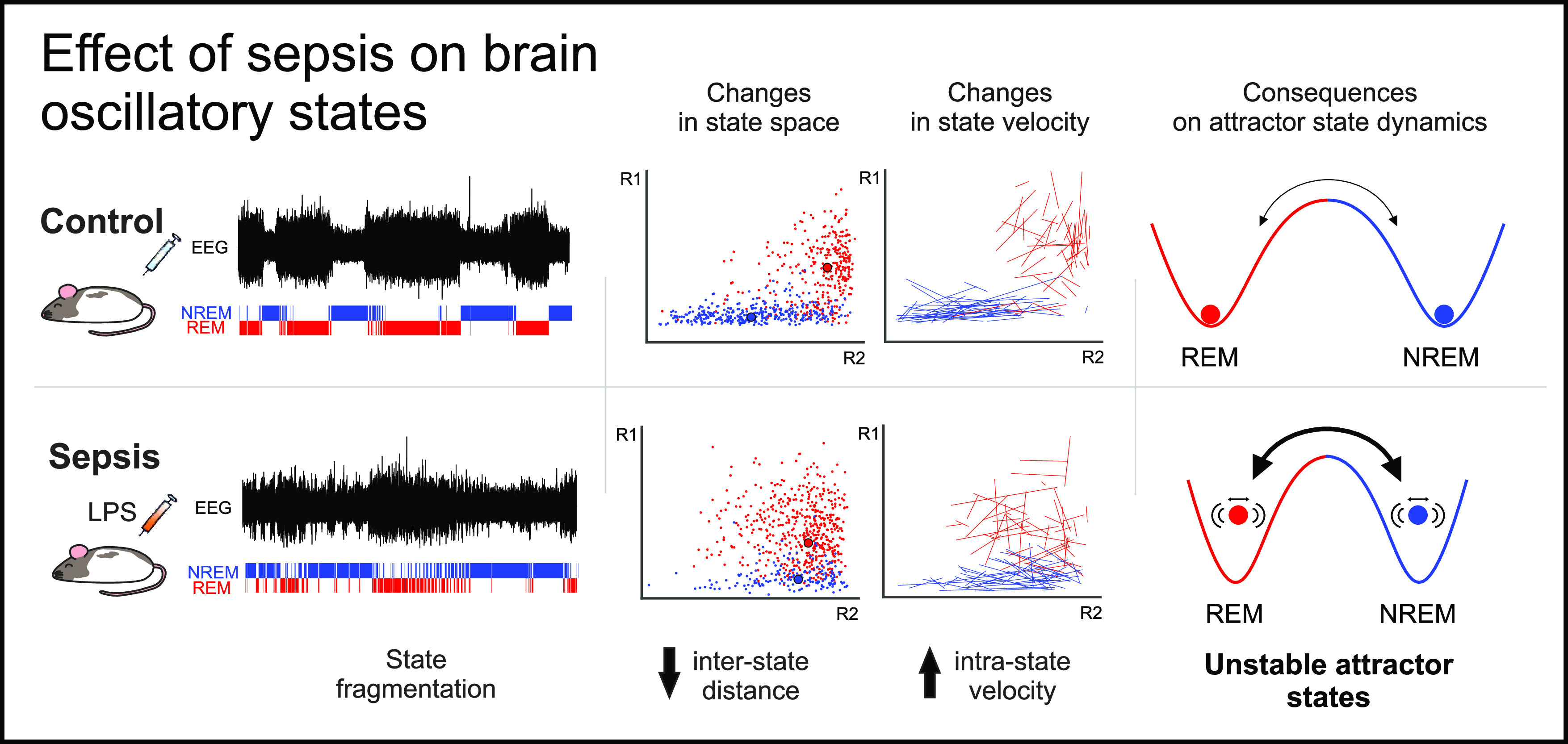

## Significance Statement

Sepsis-associated encephalopathy (SAE) is a severe complication of sepsis that leads to sleep and cognitive issues in sepsis survivors. Electrophysiological changes in brain activity may serve as early biomarkers of SAE, but also affect subsequent outcomes. Here, we investigated hippocampal oscillatory activity in a lipopolysaccharide (LPS)-mediated rat model of sepsis under urethane anesthesia, with a focus on state transition dynamics. We observed increased similarity between rapid eye movement (REM)-like and nonrapid eye movement (NREM)-like states and reduced within-state stability, as a possible cause of sepsis-related sleep fragmentation. Inability to maintain stable vigilance states not only disrupts sleep and its restorative functions, but may also directly affect cognitive functions.

## Introduction

Sepsis, a state of dysregulated host response against an infection, affects around 30 million people annually with 33% mortality (Annane and Sharshar, 2015; Rhee et al., 2017). The brain is among the first impacted organs: ∼70% of sepsis patients suffer from sepsis-associated encephalopathy (SAE) early during the disease (Czempik et al., 2020). Twenty to 25% of survivors experience postsepsis syndrome, which, besides other organ systems, often affects the brain as well (Chavan et al., 2012; Annane and Sharshar, 2015), with a wide spectrum of signs like sleep disturbances, concentration impairment, cognitive deterioration, or psychiatric conditions.

Dysregulated sleep is one of the dominant neurologic signs in intensive care unit patients during the acute sepsis (Richards and Bairnsfather, 1988; Gofton and Young, 2012). Sleep during sepsis is often markedly discontinuous and interrupted by frequent awakening (Weinhouse and Schwab, 2006; Boyko et al., 2017). The cases where cumulative sleep time was not reduced, duration of continuous sleep periods was severely shortened, impairing sleep quality and physiological functions of the sleep-related machinery (Opp, 2005; Tononi and Cirelli, 2014; Dzierzewski et al., 2020; Zamore and Veasey, 2022).

Sleep fragmentation has been described in humans and animal models of generalized inflammation or sepsis (Weinhouse and Schwab, 2006; Baracchi et al., 2011). Fragmented sleep is considered an important factor in the development of other neurologic signs in both acute systemic inflammation and chronic postsepsis syndrome (Song et al., 2021). In the acute phase, it further promotes patients’ deterioration across multiple systems by preventing efficient tissue restoration or deepening the immune dysfunction, but in case of post-sepsis syndrome, sleep fragmentation may be linked to cognitive function, mental stability, and fatigue (Sonneville et al., 2013; Spira et al., 2014; Song et al., 2021).

Besides disrupted sleep-wake rhythmicity, sepsis patients suffer from a variety of pathologic brain activity patterns, including electrographic seizures and periodic discharges and other changes in spectral and oscillatory activity. Mortality and severe disability were directly proportional to electrographic abnormalities, stressing the importance of early monitoring of brain activity (Oddo et al., 2009). Additionally, increased slow wave activity and decreased α power in the electroencephalogram (EEG) were associated with early mortality and predicted the occurrence of delirium in septic shock patients (Hosokawa et al., 2014; Azabou et al., 2015; Kinoshita et al., 2021).

These findings were partly confirmed in animal SAE models. In a rat caecal-ligation-and-puncture model, sepsis caused acute sleep fragmentation, suppression of rapid eye movement (REM) sleep and an increase in the overall amount of dark phase non-rapid eye movement (NREM) sleep (Baracchi et al., 2011). Interestingly, slow wave activity was reduced during the periods of increased NREM sleep. A different study in anesthetized rats using lipopolysaccharide (LPS)-induced sepsis found reduced power in the 8- to 13-Hz range, with no effects on δ power (Semmler et al., 2008). Under non-septic conditions, low-dose LPS modulates sleep causing NREM fragmentation in rats and increased NREM slow wave activity while suppressing REM sleep (Lancel et al., 1995). Similar effects of mild inflammation caused by LPS or Lipid A on sleep states have been observed in rabbits (Krueger et al., 1986). Other studies in rats confirmed that low doses of LPS cause NREM discontinuity, but showed reduced NREM slow wave activity instead (Kapás et al., 1998).

Apart from affecting overall sleep architecture, LPS has region-specific effects on brain oscillatory activity. Mamad et al. (2018) observed acute slowing of hippocampal θ and increased hippocampal δ frequency and power in awake rats. However, prefrontal cortex showed no changes in δ frequency, but displayed reduced θ frequencies and power instead. These varying consequences of LPS on δ power in NREM could be species-specific or dose-specific and depend on time of injection and recorded brain region.

Electrophysiological changes of brain activity in sleep could serve as a potential early biomarker of sepsis and its related outcomes. Here, we aim to understand the dynamics of REM-like and NREM-like states and their transitions during severe acute systemic inflammation caused by a high dose of LPS (10 mg/kg). We used urethane anesthesia as a model that mimics the unconscious state of sleep and produces sleep-like REM and NREM brain activity patterns, without any awakening (Clement et al., 2008). Local field potentials (LFPs) from the CA3 region of hippocampus were recorded in saline-injected and LPS-injected rats. LPS injection resulted in profound shortening of REM-like and NREM-like states. Additionally, spectral similarity between the states increased, particularly in lower frequencies (1–9 Hz). These spectral changes, leading to higher brain state similarity, might be an important contributing factor to their instability and thus to sepsis-related sleep disturbances and associated deficits in cognitive function.

## Materials and Methods

### Animals

Twelve adult male Long–Evans rats weighing 400–500 g were used. All methods were conducted in accordance with relevant guidelines and regulations. All protocols were approved by the Ethical Committee of the Ministry of Education, Youth and Sports of the Czech Republic (approval no. MSMT-12084/2019) according to the Guide for the Care and Use of Laboratory Animals (Protection of Animals from Cruelty Law, Act No. 246/92, Czech Republic) and in accordance with ARRIVE guidelines. The animals were housed individually in transparent polycarbonate cages and were kept in a 12/12 h light/dark cycle with food and water *ad libitum*. Experiments were conducted during the light phase.

### Experimental setup

**Figure 1.**
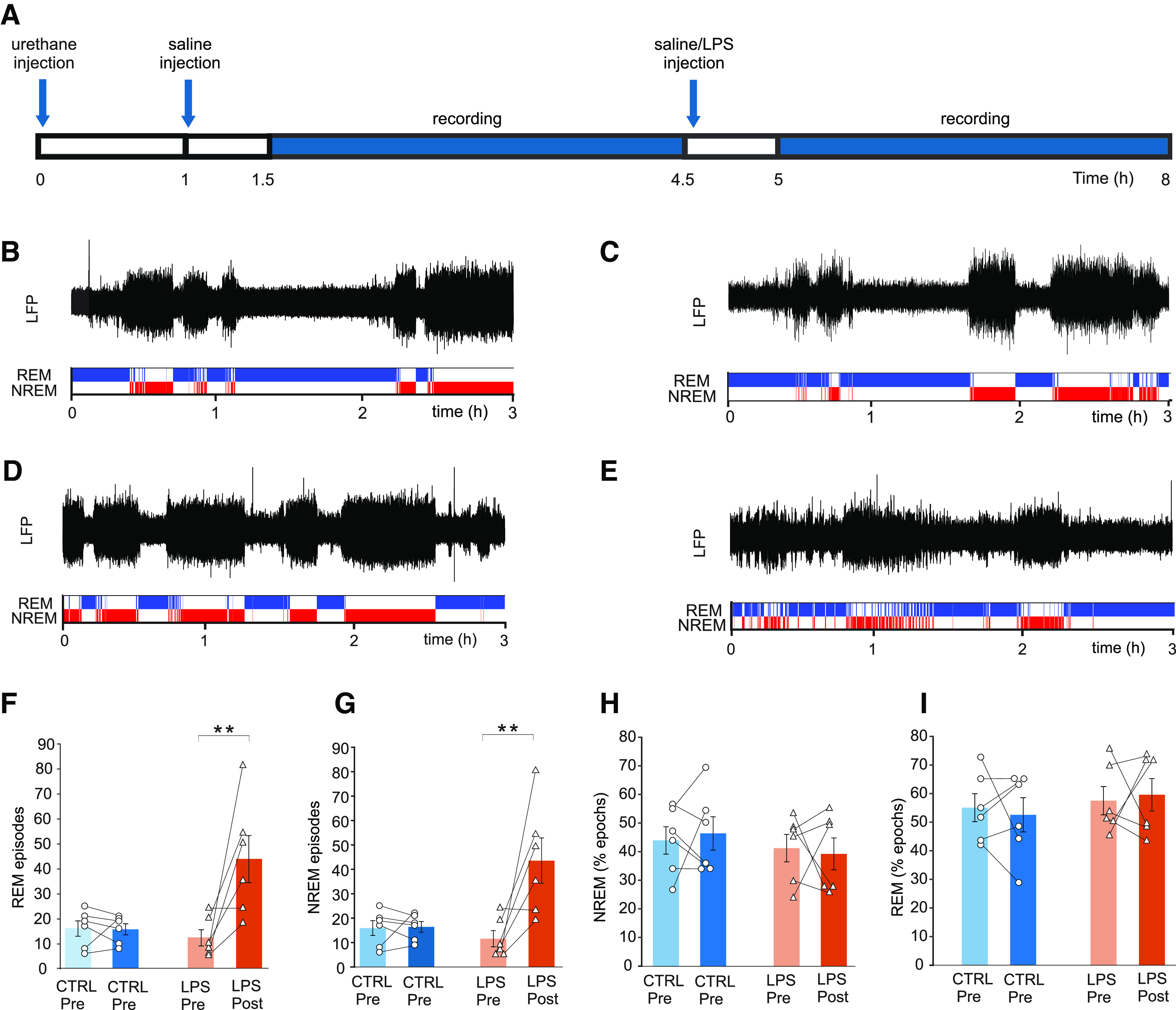
LPS causes sleep state fragmentation in rats without affecting the total time spent in either state. ***A***, Experimental design. Urethane anesthetized rats were injected with saline and baseline LFP was recorded for 3-h, followed by a single dose of LPS (LPS, *n* = 6) or saline (CTRL, *n* = 6) and another a 3-h recording. ***B–E***, Representative traces and hypnograms showing REM in blue and NREM in red. Sleep states were long and stable before (***B***) or after saline injection (***C***) in controls (CTRL) and before LPS injection (***D***) in the LPS group. After LPS injection (***E***), sleep states were heavily fragmented. ***F***, Number of REM episodes before and after injection. Each data point shows the number of episodes in individual CTRL and LPS animals before and after injection (CTRL, *n* = 6; LPS, *n* = 6). Bars show mean ± SEM, **p < 0.01. ***G***, Number of NREM episodes in CTRL and LPS animals before and after injection. **p < 0.01. ***H***, Total time spent in REM as percentage of recording time in CTRL and LPS animals before and after injection. ***I***, Total time spent in NREM as percentage of recording time in CTRL and LPS animals before and after injection.

To investigate the effects of acute systemic inflammation, two groups of rats were used (LPS and CTRL, *N* = 6 each; Fig. 1*A*). One hour after induction of urethane anesthesia, rats in both groups were injected with sterile saline (2 ml/kg, i.p.). Then, baseline hippocampal activity was recorded for 3 h. After this, rats in the LPS group were injected intraperitoneally with 10 mg/kg lipopolysaccharide (LPS) and were recorded for another 3 h. Rats in the CTRL group received a second saline injection instead. After the second recording period, blood serum samples and brains were collected for IL-1β quantification and histologic verification of electrode placement.

All animals survived until the end of the recording period. One rat in the LPS group died after the 3-h post-LPS recording period was over, just before perfusion.

### Electrode implantation surgery

Local field potentials were recorded from the rats implanted with eight independently movable tetrodes in the CA3 region of the hippocampus. Each tetrode consisted of four twisted 17-μm polyimide-coated platinum-iridium wires coated with platinum to reduce the impedance to 120–200 kΩ at 1 kHz.

First, rats were anesthetized using a mixture of ketamine (Narkamon, 100 mg/kg, i.p.) and xylazine (Rometar, 10 mg/kg, i.p.) and 1.5–2% isoflurane in O_2_. They were fixed in a stereotaxic frame and body temperature was maintained at 37°C using a heating pad. An incision was made in the scalp to expose the skull, after which a craniotomy was made over the dorsal hippocampus (3.8 mm caudal, 3.2 mm lateral of bregma). After removal of the dura mater, the tetrode bundle was carefully lowered into the cortex. Individual tetrodes were slowly lowered into CA3 over the course of a two-week recovery period. The hyperdrive was fixed to the skull using dental acrylic and stainless-steel screws. One screw, located above the frontal cortex, served as a reference. Rats were given carprofen (Rimadyl, 5 mg/kg, s.c.) and Marbofloxacin (Marbocyl, 5 mg/kg, s.c.) during recovery. Rats were used in behavioral experiments for two to three weeks after surgery, before the experiments described in the current paper.

### Recording procedure

Rats were anesthetized for recording using urethane (1.5 g/kg, i.p.; Sigma). One hour after urethane injection, hippocampal local field potentials were amplified using an Intan RHD2132 headstage amplifier, digitized, and recorded using an OpenEphys recording system at a sampling rate of 2000 Hz and a high-pass filter set at 1 Hz (Siegle et al., 2017).

### Data processing and analysis

#### Signal processing

For vigilance state classification and later state-space analysis, a sliding window FFT analysis (2-s window, 1-s step) was performed on separately for all recorded channels using Welch’s method in MATLAB 2014b (Hamming window, 50% overlap, 0.25-Hz resolution), yielding one epoch per second. Then, spectral ratios were calculated for overall power in two overlapping frequency ranges for each time window. Ratio 1 was calculated as R1 = (1–2 Hz)/(1–9 Hz) and Ratio 2 was calculated as R2 = (1–15 Hz)/(1–45 Hz). Frequency ranges were chosen based on literature and on the approximate frequencies of δ and θ peaks in our recorded baseline spectra (Gervasoni et al., 2004; Diniz Behn et al., 2010). Normalized signal amplitude was calculated using the same sliding window approach, where mean absolute signal amplitude was calculated for each window and normalized to mean absolute signal amplitude for the entire recording. The resulting power and amplitude time series for all channels were then combined into a single time series per rat for each of these variables using principal component analysis. The first principal component was used for state space analyses, as described previously (Gervasoni et al., 2004; Diniz Behn et al., 2010). The Ratio 1 and Ratio 2 time series were generally well represented by this principal component, which explained 91.83 ± 1.26% and 91.83 ± 0.9% of variance in both ratios at all time points for R1 and R2, respectively. Epochs that contained artefacts were excluded from further analysis (0.18 ± 0.06% of recording time). State labels for these epochs were set to be identical to the preceding, artifact-free epoch before smoothing.

#### Vigilance state classification and episode detection

Brain activity under urethane anesthesia showed two distinct alternating states: a NREM-like state that was dominated by low-frequency, high-amplitude waves, and a REM-like state with faster activity and a lower signal amplitude. Epochs were automatically classified as belonging to one of these states based on the calculated principal component values for R1, R2, and amplitude using k-means clustering. Inclusion of the amplitude parameter in the clustering procedure ensured reliable state identification, even when spectral ratios were affected by experimental treatment.

After initial clustering, ultra-short periods of NREM-like or REM-like activity were removed: a state transition was only considered if the first epoch of the new state was followed by at least three more epochs of the same state. Otherwise, the epoch was labeled as belonging to the preceding state. This smoothing procedure ensured that any unrealistically short, artefactual state changes caused by normal within-state signal variability were removed.

NREM-like and REM-like episodes were calculated as periods of each state that were at least 10 epochs long, and were followed by at least 10 epochs of the other state. Short episodes were considered as 20–120 s long, whereas episodes of 600 s or more were considered long. Epoch-to-epoch transition probability was calculated based on the percentage of epoch that was followed by the same or a different state. Episode transitions were characterized by fitting a logistic curve using MATLAB’s fit function with the following equation f(10) = offset + (range/(1 + e^–slope*x^)). Curves were fit to a 30-s period of LFP trace centered on the episode state transition after smoothing using a 5-s window moving average.

#### State-space analysis

Inflammation-related spectral changes were analyzed in a two-dimensional state-space based on R1 and R2. This type of analysis may reveal within and between state dynamics that are not captured using less sensitive single-band approaches (Gervasoni et al., 2004; Diniz Behn et al., 2010). Cluster positions were defined using the median pc1R1 and pc1R2 values for each state.

Within-state jitter was analyzed using epoch-to-epoch distances in state-space. Jitter was defined as distance between two subsequent epochs. As such, overall jitter was calculated as j = √((pc1R1*_n+1_*– pc1R1*_n_*)^2^ + (pc1R2*_n+1_*– pc1R2*_n_*)^2^), and jitter along a single dimension such as R1 simply as j*_R1_* = pc1R1*_n+1_* – pc1R1*_n_*

#### Analysis of periodic and aperiodic spectrum components

To further investigate how power spectrum changes lead to the observed state-space effects, aperiodic and periodic components of the power spectrum were parametrized using the FOOOF algorithm (v. 1.0.0) in Python 3.7 (Haller et al., 2018; Donoghue et al., 2020). First, average power spectra for each state were calculated from the sliding window FFT described earlier. One representative channel was analyzed per rat and the same channel was used for the preinjection and postinjection time points. For each spectrum, the frequency range from 1 to 45 Hz was used with the following algorithm settings: peak width limits 0.5 and 12 Hz, maximum number of peaks 6, minimum peak height 0.2, peak threshold 2.0, and aperiodic mode: knee. Broad band γ oscillations in the 15- to 30-Hz frequency range are not adequately captured by these settings. For these oscillations, the calculated aperiodic component was subtracted from the power spectrum first, after which peaks were fit with a minimum peak height 0.1, peak threshold 2.0, minimum peak width 0.5, but without a maximum peak width.

Aperiodic spectral components were modelled using the following aperiodic fit AP(f) = 10^b^ × (1/(k+f^χ^)) fit (AP) where f is frequency, b is offset, k is the knee parameter, and χ is the spectrum slope. The knee parameter represents the bending point where the aperiodic fit transitions from horizontal to negatively sloped. Knee frequency is dependent on the value of k and spectrum slope χ and was calculated as k_freq_ = k^(1/χ)^. Periodic components of the spectrum, representing putative oscillations, were modelled as Gaussian curves over and above the aperiodic background spectrum. Each oscillation has a center frequency (c), peak width (w), and center peak height (a), yielding the following for each frequency f G(f) = a × exp(–(f – c)^2^/(2 × w^2^)).

#### Statistics

Statistical analyses were performed using JASP 0.13.1 and MATLAB 2014b. Time and treatment group effects were analyzed using repeated measures (rm)ANOVA. Where sphericity assumptions were violated, the Greenhouse–Geisser correction was used to adjust *p*-values. Within-group changes in oscillatory and aperiodic activity were analyzed using Wilcoxon’s signed-rank test. As these tests were applied to different aspects of the same peaks or background spectra, Bonferroni correction was used to adjust *p*-values for the three comparisons per set of tests. Bootstrapped correlations using Spearman’s method and mean ± SD was reported for the obtained ρ values.

### Cytokine quantification and histology

After recording, blood samples were collected and serum was separated by centrifugation at 1000 × *g* for 10 min. Levels of IL-1β were quantified using an ELISA kit (RAB0277, Sigma) according to manufacturer’s protocol.

Then, rats were killed using an overdose of sodium pentobarbital (50 mg/kg) and perfused transcardially with ringer solution followed by 4% paraformaldehyde in phosphate-buffered saline. Brains were collected and cut in 50-μm-thick coronal sections. Electrode placement was verified using Nissl staining. LFP traces from electrodes outside of hippocampal CA3 were excluded from analysis.

### Data availability

Data are available on request from the corresponding author.

## Results

### LPS raises serum IL-1β levels

IL-1β levels were measured to confirm systemic inflammation. Serum IL-1β concentrations in LPS-injected rats were at least three times higher than controls (398 ± 32.24 vs 122 ± 63.64 pg/ml, *n* = 6, independent sample *t* test *t*_(10)_ = 3.88, *p* < 0.01).

### LPS injection causes altered sleep state structure

Rats showed two distinct brain activity patterns under urethane anesthesia: a NREM-like state dominated by high amplitude slow waves, and a REM-like state with low-amplitude, higher frequency activity (Fig. 1*B–E*). The states were long and stable at baseline and in saline-injected controls (Fig. 1*B–D*), but much shorter after LPS injection (Fig. 1*E*).

Fragmentation manifested as more REM episodes (rmANOVA; time effect: *F*_(1,10)_ = 8.357, *p* = 0.016; group effect: *F*_(1,10)_ = 5.277, *p* = 0.044; group × time interaction: *F*_(1,10)_ = 8.719, *p* = 0.014; Fig. 1*F*), and also NREM episodes (rmANOVA; time effect: *F*_(1,10)_ = 8.357, *p* = 0.016; group effect: *F*_(1,10)_ = 5.277, *p* = 0.044; group × time interaction: *F*_(1,10)_ = 8.719, *p* = 0.014; Fig. 1*G*).

Despite increased episode numbers, time spent in NREM (rmANOVA; time effect: *F*_(1,10)_ = 0.002, *p* = 0.96; group effect: *F*_(1,10)_ = 0.77, *p* = 0.39; group × time interaction: *F*_(1,10)_ = 0.199, *p* = 0.66) and REM (rmANOVA; time effect: *F*_(1,10)_ = 0.002, *p* = 0.96, group effect: *F*_(1,10)_ = 0.77, *p* = 0.39; group × time interaction: *F*_(1,10)_ = 0.199, *p* = 0.66) were unaltered by LPS injection (Fig. 1*H*,*I*).

**Figure 2.**
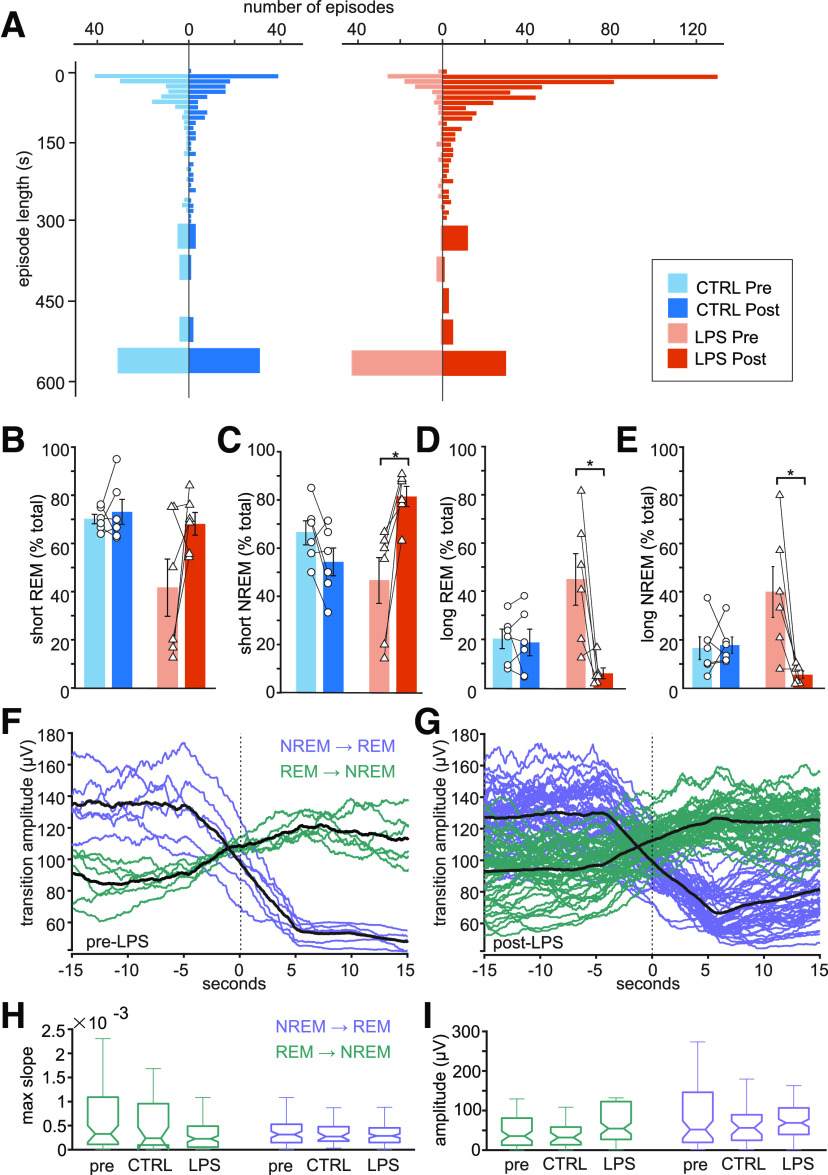
LPS leads to an increase in short and a decrease in long sleep state episodes with normal transitions. ***A***, Histograms depicting the overall distribution of episode lengths in CTRL (left) and LPS rats (right), before and after injection. Each bar in the histogram shows the number of episodes per 10- or 50-s bin. ***B***, ***C***, Percentages of short 20- to 120-s episodes of REM (***B***) or NREM (***C***) in CTRL and LPS rats, before and after injection (*n* = 6 per group). ***D***, ***E***, Percentages of long ≥600-s episodes of REM (***D***) or NREM (***E***) in CTRL and LPS rats, before and after injection. Bar graphs show mean ± SEM, *p < 0.05. ***F***, ***G***, Smoothed EEG traces for state transitions for a representative animal before (***F***) and after LPS injection (***G***). Mean traces are shown in black and individual transitions in purple and green. ***H***, Box plots showing medians and quartiles for maximum transition slope for all NREM-to-REM and REM-to-NREM transitions in all preinjection recordings (*N* = 80 and *N* = 79, resp., 12 rats), postinjection in controls (CTRL, *N* = 37 and *N* = 34, resp., 6 rats), and after LPS injection (*N* = 123 and *N* = 116, resp., 6 rats). ***I***, Box plots showing medians and quartiles for transition amplitudes for all NREM-to-REM and REM-to-NREM transitions in all preinjection recordings (*N* = 80 and *N* = 79, resp., 12 rats), postinjection in controls (CTRL, *N* = 37 and *N* = 34, resp., 6 rats), and after LPS injection (*N* = 123 and *N* = 116, resp., 6 rats).

Extensive sleep instability was further apparent in episode length distribution (Fig. 2*A*). At baseline and after saline injection rats had few short episodes (20–120 s) and a relatively many long episodes (≥600 s). After LPS injection, however, brain activity patterns consisted of many short episodes and only a few long ones. Relative amounts of short episodes were significantly higher in NREM after LPS injection (rmANOVA, time effect: *F*_(1,10)_ = 4.67, *p* = 0.05, group effect: *F*_(1,10)_ = 0.25, *p* = 0.62, group × time interaction: *F*_(1,10)_ = 19.78, *p* < 0.01), but not in REM (rmANOVA, group effect: *F*_(1,10)_ = 6.58, *p* = 0.02, time effect: *F*_(1,10)_ = 4.01, *p* = 0.07, group × time interaction: *F*_(1,10)_ = 2.56, *p* = 0.14; Fig. 2*B*,*C*). *Post hoc* analyses showed a significant increase in the percentage of short NREM episodes in the LPS group postinjection (81.44 ± 4.58% of NREM episodes vs 46.59 ± 10.37% preinjection) and a significant baseline difference in short REM episodes between controls (70.12 ± 1.94%) and the LPS group (41.52 ± 11.88%). Changes in long episodes were the opposite to those in short episodes (Fig. 2*D*,*E*). The occurrence of long NREM episodes was significantly reduced after LPS injection (5.90 ± 1.55%), compared with baseline (39.97 ± 10.48%), although episode numbers are generally low (rmANOVA, time-effect: *F*_(1,10)_ = 8.28, *p* = 0.01, group × time interaction: *F*_(1,10)_ = 9.40, *p* = 0.01, group effect: *F*_(1,10)_ = 0.79, *p* = 0.39). Effects on long REM episodes were similar (rmANOVA, time effect: *F*_(1,10)_ = 9.78, *p* = 0.01, group × time interaction: *F*_(1,10)_ = 8.39, *p* = 0.01, group effect: *F*_(1,10)_ = 0.89, *p* = 0.36). *Post hoc* analysis showed significantly fewer long REM episodes in the LPS group post-injection (6.51 ± 2.12%) compared with pre-injection (44.16 ± 10.42%). These results point to instability in both the REM and NREM state.

State instability was also apparent on the epoch-to-epoch level, where the probability of state transition from one epoch to the next was similarly increased for NREM-to-REM transitions (rmANOVA; time effect: *F*_(1,10)_ = 16.72, *p* < 0.01; group effect: *F*_(1,10)_ = 4.61, *p* = 0.06; group × time interaction: *F*_(1,10)_ = 10.53, *p* < 0.01) and REM-to-NREM transitions (rmANOVA; time effect: *F*_(1,10)_ = 16.12, *p* < 0.01; group effect: *F*_(1,10)_ = 4.77, *p* = 0.05; group × time interaction: *F*_(1,10)_ = 10.68, *p* < 0.01). *Post hoc* analysis showed significantly increased transition probabilities in both directions after LPS injection (NREM-to-REM: 0.17 ± 0.05% pre vs 0.62 ± 0.10% post; REM-to-NREM: 0.17 ± 0.05% pre vs 0.62 ± 0.09% post). State transition probabilities remained at baseline levels in controls (NREM-to-REM: 0.22 ± 0.05% vs 0.27 ± 0.05%; REM-to-NREM: 0.27 ± 0.05% vs 0.27 ± 0.05%)

Although a higher number of state transitions was found after LPS, transition characteristics were not significantly different from those in controls or at baseline (Fig. 2*F–I*). Thus, LPS injection leads to instability of NREM and REM to a similar degree, without affecting time spent in either state or transition characteristics between the states.

### LPS leads to increased spectral similarity between REM and NREM

**Figure 3.**
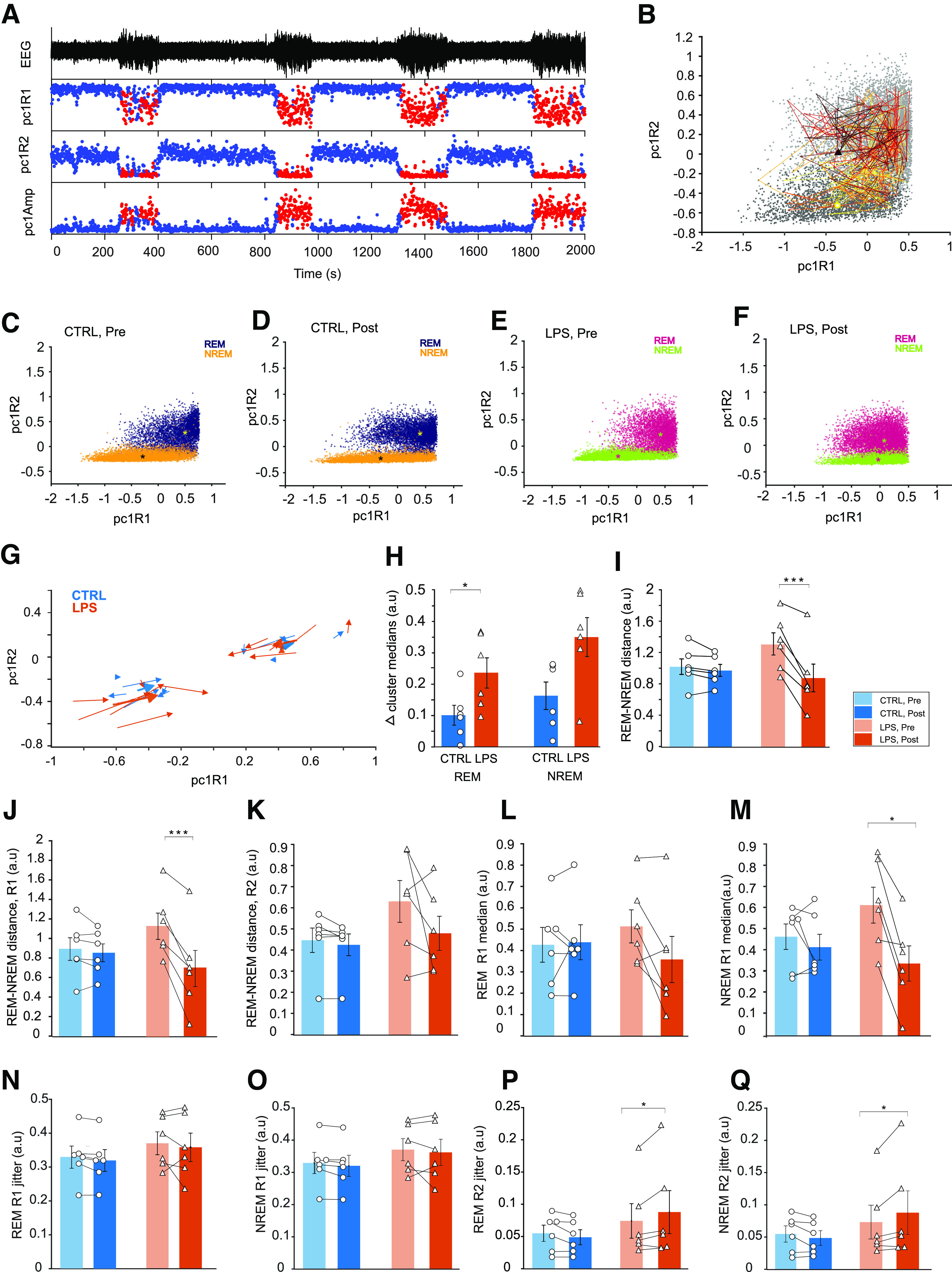
LPS leads to reduced cluster distance and increased jitter. ***A***, First principal components of spectral ratios R1 and R2 in time, as well as the amplitude component used for state classification, with the accompanying EEG trace. Epochs classified as NREM are shown in red, epoch classified as REM in blue. ***B***, Example of a 400-s-long trajectory in 2-D state space. The shown trace starts in the NREM cluster (circle) and eventually ends in the REM cluster after covering much of the recording’s state-space. Epochs that are not part of this trajectory are shown in gray. C-F. Scatter plots showing REM and NREM epochs in 2-D state space in CTRL (***C***, ***D***) and LPS (***E***, ***F***) groups, before and after injection. The asterisk marks the cluster median. ***G***, Quiver plot showing the directional shift of the REM and NREM cluster medians before and after injection in CTRL (blue) and LPS animals (orange). The starting point of each arrow shows cluster medians before the injection, and the arrow head the medians after injection. ***H***, Shifts in cluster medians in REM and NREM. Change in cluster position after treatment in individual CTRL or LPS animals are shown. Bars show mean ± SEM. ***I***, Overall 2-D distance between REM and NREM clusters in CTRL and LPS animals before and after injection. *p < 0.05, ***p < 0.001. ***J***, ***K***, Distance between REM and NREM cluster medians along R1 (***J***) or R2 (***K***) in CTRL (circles) and LPS (triangles) animals before or after injection. Bars represent mean ± SEM, ***p < 0.001. ***L–M***, R1 medians in REM (***L***) and NREM (***M***) in CTRL and LPS animals before or after injection. Bars represent mean ± SEM, **p < 0.05, ***p < 0.001. ***N***, ***O***, Median jitter values in REM (***N***) and NREM (***P***) along R1 in CTRL and LPS animals before or after injection. Bars represent mean ± SEM. ***P***, ***Q***, Median jitter in REM (***P***) and NREM (***Q***) along R2 in CTRL and LPS animals before or after injection. Bars represent mean ± SEM, *p < 0.05.

Changes in spectral characteristics were assessed based on spectral power Ratio 1 (R1, 1–2 /1–9 Hz) and Ratio 2 (R2, 1–15/1–45 Hz), which are distinct in the two observed states (Fig. 3*A*,*B*). State-space analysis based on these ratios resulted in two distinct state clusters of epochs in the control and LPS group at baseline and after injection (Fig. 3*C–F*).

After LPS injection (Fig. 3*F*), the location of the NREM and REM clusters within state-space shifted, resulting in a decreased intercluster distance. The magnitude and direction of the changes in cluster location after LPS injection and in controls is shown in Figure 3*G*.

The centroid of REM cluster shifted significantly in LPS group compared with controls (*t*_(10)_ = 2.33, *p* = 0.04; Fig. 3*H*), with a mean vector direction toward the origin and magnitudes of 0.23 ± 0.05 a.u. and 0.10 ± 0.03 a.u., respectively. NREM cluster shifts were not significantly different in controls (0.16 ± 0.04 a.u.) and in LPS rats (0.35 ± 0.06 a.u., *t*_(10)_ = 1.45, *p* = 0.17), because of higher variability. The mean NREM vector was directed away from origin, toward the REM cluster.

As a result of the observed shifts in cluster medians, the distance between the two clusters decreased (Fig. 3*I*). We calculated the distance between REM and NREM clusters before and after injection in control and LPS groups (rmANOVA, time effect: *F*_(1,10)_ = 26.47, *p* < 0.001, group × time interaction *F*_(1,10)_ = 17.09, *p* < 0.01, group effect: *F*_(1,10)_ = 0.3, *p* = 0.59). *Post hoc* analysis showed significantly smaller distances between REM and NREM clusters in the LPS group postinjection (0.87 ± 0.19 a.u.) compared with baseline (1.30 ± 0.15 a.u.). Decreased interstate cluster distances indicate increased spectral similarity between the states.

### State similarity in low spectrum frequencies

Decreased distance between REM and NREM clusters could be the result of changes in R1, R2, or in both. By decomposing the two-dimensional intercluster distance into R1 and R2 components, we determined the spectral ratio most affected by LPS. Distance between REM and NREM clusters was significantly decreased along R1 (rmANOVA; time effect *F*_(1,10)_ = 39.26, *p* < 0.001, group effect *F*_(1,10)_ = 0.03, *p* = 0.84, group × time interaction, *F*_(1,10)_ = 27.55, *p* < 0.001). *Post hoc* analysis showed that the distance between clusters was significantly smaller in the LPS group postinjection (0.69 ± 0.20 a.u.) compared with baseline (1.13 ± 0.14 a.u.; Fig. 3*J*). By contrast, there was no significant change in the distance between the clusters along R2 (rmANOVA; time effect, *F*_(1,10)_ = 2.85, *p* = 0.12, group × time interaction, *F*_(1,10)_ = 1.59, *p* = 0.23, group effect, *F*_(1,10)_ = 1.64, *p* = 0.22; Fig. 3*K*).

Hence, LPS-mediated spectral similarity between REM and NREM is resulting mainly from changes in the 1- to 9-Hz frequency range.

### NREM contributes more to spectral similarity in lower frequencies than REM

Reduced distance between REM and NREM clusters along R1 may be state-specific or both states could contribute. In REM, we observed no significant changes in median R1 values (rmANOVA; time effect: *F*_(1,10)_ = 3.16, *p* = 0.10, group effect: *F*_(1,10)_ = 0.0002, *p* = 0.98, time × group interaction: *F*_(1,10)_ = 4.31, *p* = 0.06; Fig. 3*L*).

NREM R1 values were significantly affected, however (rmANOVA, time effect: *F*_(1,10)_ = 15.02, *p* < 0.01), time × group interaction: *F*_(1,10)_ = 7.27, *p* = 0.02, group effect (*F*_(1,10)_ = 0.13, *p* = 0.72). *Post hoc* analysis showed a significant decrease in R1 medians in the LPS group postinjection (0.34 ± 0.09 a.u.) compared with baseline (0.61 ± 0.09 a.u.; Fig. 3*M*).

### LPS caused increased within-state instability in REM and NREM

The observed state fragmentation and altered REM-NREM dynamics could be caused by inflammation-related state instability. Here, we used distance between subsequent epochs in state-space as a measure of within-state jitter or instability.

Jitter along R1 was not significantly affected by LPS injection in either REM (rmANOVA; time effect: *F*_(1,10)_ = 1.20, *p* = 0.29; group effect: *F*_(1,10)_ = 0.80, *p* = 0.39; time × group interaction: *F*_(1,10)_ = 0.01, *p* = 0.92; Fig. 3*N*), or NREM (time effect: *F*_(1,10)_ = 0.92, *p* = 0.36; group effect: *F*_(1,10)_ = 0.86, *p* = 0.37; time × group interaction: *F*_(1,10)_ = 0.0005, *p* = 0.98; Fig. 3*O*).

However, jitter along R2 was significantly increased in both REM and NREM after LPS injection. In REM, *post hoc* analyses showed significantly higher jitter in LPS rats after injection (0.087 ± 0.03 a.u.) compared with baseline (0.074 ± 0.03 a.u.; rmANOVA, time × group interaction: *F*_(1,10)_ = 7.93, *p* = 0.01, group effect *F*_(1,10)_ = 0.96, *p* = 0.34, time effect: *F*_(1,10)_ = 1.17, *p* = 0.30; Fig. 3*P*). Effects on R2 jitter in NREM were similar (rmANOVA, time × group interaction: *F*_(1,10)_ = 7.15, *p* = 0.02, group effect: *F*_(1,10)_ = 0.96, *p* = 0.34, time effect: *F*_(1,10)_ = 1.17, *p* = 0.30; Fig. 3*Q*), although *post hoc* analyses showed no significant differences.

### Effects of LPS on periodic and background power spectrum components

**Figure 4.**
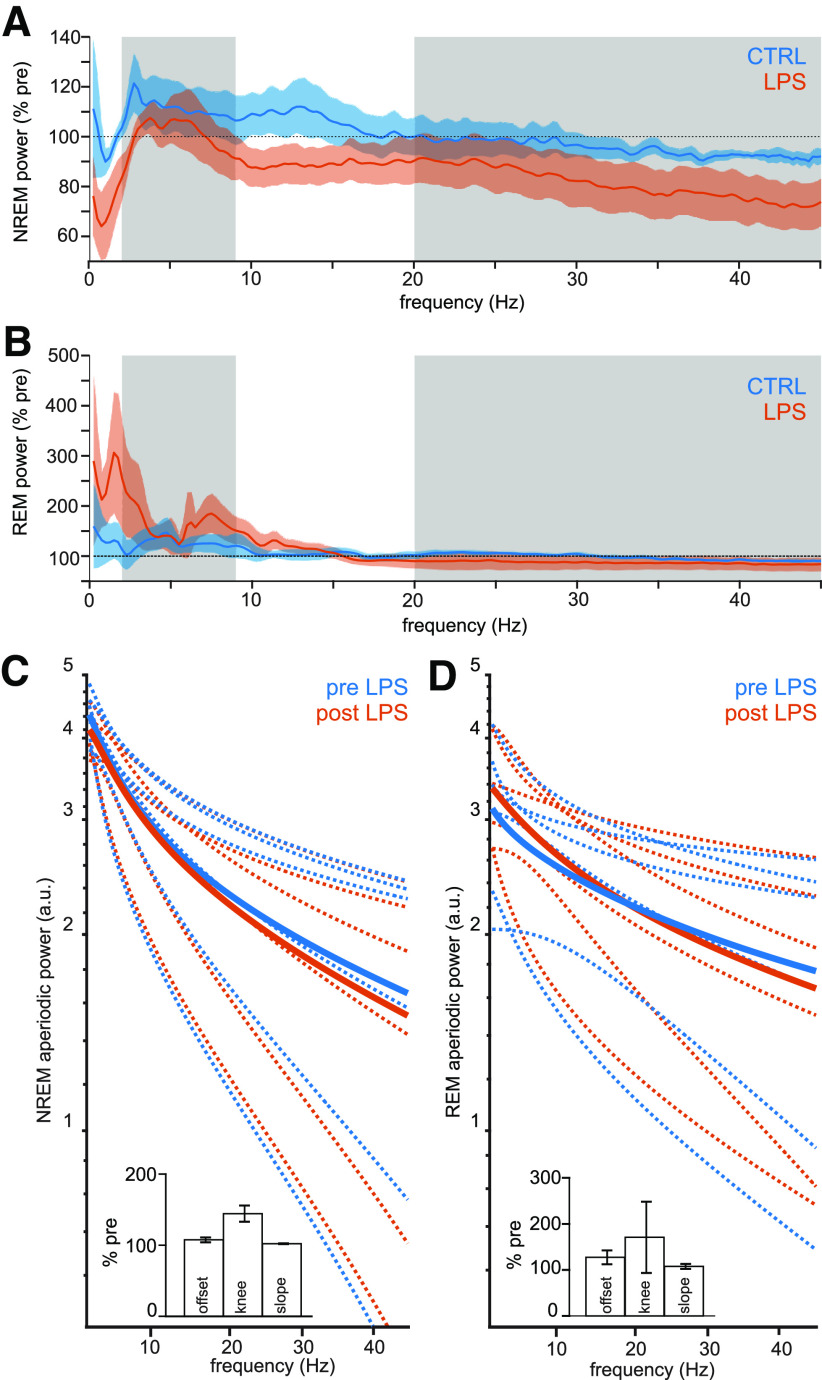
LPS-related changes in NREM and REM power spectra. ***A***, NREM power spectra of control (blue) and LPS-injected (orange) rats as percentage of preinjection baseline. Gray areas indicate limits of the spectral ratios used for state-space analysis. ***B***, REM power spectra of control (blue) and LPS-injected (orange) rats as percentage of preinjection baseline. Gray areas as in ***A***. ***C***, ***D***, Aperiodic component of the LFP spectrum before and after LPS injection in NREM (***C***) and REM (***D***). Solid lines show group average, dotted lines show individual animals. Insets show changes in offset, slope, and knee frequency, which define the aperiodic spectral component for each state. Bars show mean ± SEM.

To investigate possible sources of the changes in R1, power spectra were analyzed for representative channels. NREM spectra showed markedly reduced power below 3 Hz in LPS-injected rats, but not controls (Fig. 4*A*). REM spectra showed the opposite effect: increased power in the <3-Hz range, as well as a smaller, variable increase in the 7- to 9-Hz range (Fig. 4*B*). Power spectra in the control group remained at baseline levels.

Post-LPS changes in spectral power could be caused by altered EEG oscillations and background (aperiodic) components of the power spectrum. To better understand post-LPS R1 in REM and NREM, aperiodic and periodic components of the power spectrum for each state were modelled separately. Overall model fits were good for NREM spectra (*R*^2^ = 0.995 ± 0.002, fit error = 0.031 ± 0.006) and REM spectra (*R*^2^ = 0.987 ± 0.008, fit error = 0.035 ± 0.007).

Like in nonanesthetized recordings (Leemburg et al., 2018), REM spectra had overall shallower slopes than NREM spectra at baseline (REM vs NREM exponents: 1.42 ± 0.28 vs 1.95 ± 0.18), and after LPS (REM vs NREM exponents: 1.67 ± 0.22 vs 2.09 ± 0.19; Fig. 4*C*,*D*)

Aperiodic NREM spectrum components were not significantly affected by LPS (Fig. 4*C*). Slopes remained at baseline levels (Wilcoxon signed-rank test, W = 21, *p* = 0.09 after Bonferroni correction), as did offsets (W = 21, *p* = 0.09 after Bonferroni correction). Knee frequencies were more variable than the other parameters and were slightly, but nonsignificantly, increased by LPS (144.39 ± 11.36%, W = 21, *p* = 0.09 after Bonferroni correction). Effects of LPS on aperiodic REM spectrum components were similar (Fig. 4*D*). Slopes were not significantly changed after LPS (W = 16, *p* = 0.94 after Bonferroni correction). Knee frequencies (W = 15, *p* = 1.31 after Bonferroni correction) and offsets were likewise unaffected (W = 16, *p* = 0.94 after Bonferroni correction).

**Figure 5.**
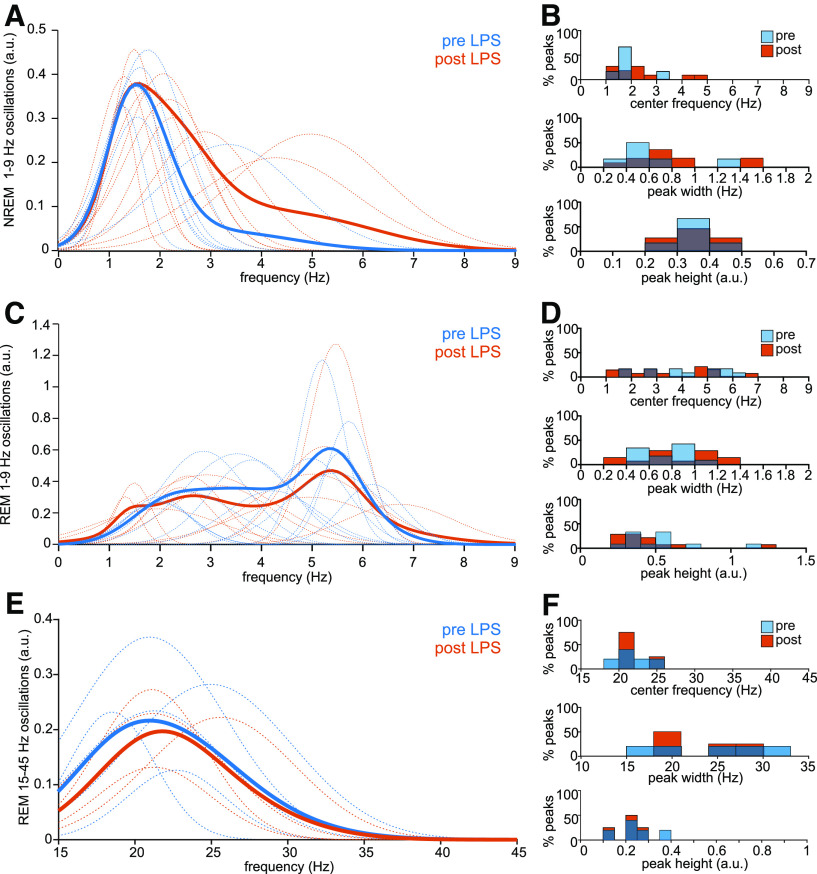
LPS-induced changes in periodic LFP activity. ***A***, NREM oscillations in the 1- to 9-Hz frequency range before (blue) and after (orange) LPS injection. Dotted lines show individual detected periodic peaks, solid lines show the mean oscillatory power. ***B***, Distribution of NREM Gaussian peak characteristics before (blue) and after (orange) the LPS injection. Shown are center frequency, peak width, and peak height in the 1- to 9-Hz frequency range. ***C***, REM oscillations in the 1- to 9-Hz frequency range prior (blue) and after (orange) the LPS injection. Dotted lines show individual detected periodic peaks, solid lines show the mean oscillatory power. ***D***, Distribution of REM Gaussian peak characteristics before (blue) and after (orange) the LPS injection. Shown are center frequency, peak width, and peak height in the 1- to 9-Hz frequency range. ***E***, REM oscillations in the 15- to 45-Hz frequency range prior (blue) and after (orange) the LPS injection. Dotted lines show individual detected periodic peaks, solid lines show the mean oscillatory power. ***F***, Distribution of REM Gaussian peak characteristics before (blue) and after (orange) the LPS injection. Shown are center frequency, peak width, and peak height in the 15- to 45-Hz frequency range.

At baseline, NREM spectra showed one major periodic component in the δ frequency range with a center frequency of 1.56 ± 0.06 Hz and peak widths between 0.2 and 0.8 Hz (Fig. 5*A*). After LPS injection, center frequencies of this oscillation increased and peak widths became more variable (Fig. 5*B*), leading to overall faster and more variable NREM δ oscillations. As such, spectral power in frequencies above 2 Hz increased, resulting in the observed R1 shift. NREM spectra did not contain other oscillatory components.

REM spectra showed multiple oscillations: a δ-like oscillation with a peak frequency in the 1- to 2-Hz range, and θ-like oscillations with a peak frequency around 5–6 Hz (Fig. 5*C*). These lower frequency oscillations remained present after LPS, but peak frequencies were lower, and more oscillations had peak frequencies below 2 Hz. Additionally, REM peak widths became more variable and overall peak heights slightly lower (Fig. 5*D*). This slowing of oscillatory components resulted in more power in the 1- to 2-Hz range and less in frequencies over 2 Hz. This effect, opposite to that found in the NREM spectrum, resulted in decreased inter-state distances between clusters in state-space along Ratio 1.

Additionally, REM spectra contained a broad band oscillation in the γ frequency range in most animals, with peak frequencies around 20–25 Hz and peak widths ranging from 15 to 32 Hz (Fig. 5*E*,*F*). These oscillations remained present after LPS, and showed no major changes from baseline.

### Relations between inflammation severity, state fragmentation, and state similarity

Overall state fragmentation was positively correlated with higher IL-1β serum concentrations at the end of the experiment (Spearman’s ρ 0.682 ± 0.202, *p* = 0.009 for all rats, Spearman’s ρ 0.736 ± 0.238, *p* = 0.058 for LPS-injected animals only, mean ± SD). Inflammation severity, rather than the septic state per se resulted in state fragmentation.

Further, increases in state fragmentation were correlated with changes in inter-state distances. That is, animals with the largest decreases in inter-state distance also showed the largest increases in state fragmentation (Spearman’s ρ 0.700 ± 0.233, *p* = 0.0188 for all rats, Spearman’s ρ 0.909 ± 0.173, *p* = 0.175 for LPS-injected animals only, mean ± SD).

## Discussion

Here, we used the lipopolysaccharide model of sepsis in rats to characterize effects of severe generalized inflammation on hippocampal oscillation state dynamics. We found profound alterations of EEG state dynamics in LPS-injected animals, mainly expressed as instability between the NREM-like and REM-like states: the states alternated considerably more frequently as the episode lengths were shortened by ≈75%. Additionally, time spent in either state was not affected by LPS, indicating that the observed effect is unlikely to be the result of selective suppression of one of the states. Instead, we identified changes in frequency characteristics of both states, resulting in their increased spectral similarity. In addition, the generalized inflammatory response led to more within-state instability marked by higher state-space jitter.

The changes in oscillatory spectra of observed states might be directly related to their fragmented dynamics. States like REM and NREM, or like sleep and waking, represent global brain activity states with attractor properties whose adequate stability is essential for maintaining their respective physiological functions (Allison and Cicchetti, 1976; Amit and Amit, 1989; Cirelli, 2006). Their relation can be illustrated as an energy landscape where the states represent local minima separated by higher energy saddles (Hopfield, 1982; Rolls, 2021). Transition from one attractor into another can be initiated by an external energy applied to the network. Under pathologic conditions however, stability might be affected by cell activity changes leading to alterations in the attractor landscape (Loh et al., 2007) . Although findings about changes in cell population activity levels across various sepsis and inflammation models are ambiguous (Riazi et al., 2010; Gullo et al., 2014; Zhang et al., 2023), increased spectral similarity between the two oscillatory states described here might represent alterations in energy landscape topology that could facilitate transitions. Increased interstate similarity combined with elevated internal noise in the complexity-reduced model might thus provide substantial knowledge in mechanistic explanation of sleep fragmentation by wakefulness observed in human SAE patients and in related animal models.

Sleep disturbances are a frequent complication of acute systemic inflammation in early-stage and developed-stage sepsis patients, and may persist in postsepsis syndrome (Richards and Bairnsfather, 1988; Weinhouse and Schwab, 2006; Weinhouse et al., 2009). In the acute stage, state fragmentation presents as many short sleep episodes that are widely distributed across the 24-h period (Baracchi et al., 2011), indicating possible disturbances in both the homeostatic and circadian sleep regulatory systems. Changes of central melatonin release rhythms (Bourne et al., 2008), as well as circadian regulation of suprachiasmatic and hypothalamic-pituitary-adrenal-axis activity have been described (Carlson et al., 2006; Cavadini et al., 2007; Beynon and Coogan, 2010). Although the molecular drivers of sleep homeostasis are not yet fully known, inflammation has been shown to affect expression molecules linked to sleep regulation, like BDNF and adenosine 2A receptors (Ohta and Sitkovsky, 2001; Schmitt et al., 2016; Pereira de Souza Goldim et al., 2020; Cui et al., 2021; Lanza et al., 2022; Reichert et al., 2022). Additionally, cytokine signaling plays an important role in regulating sleep pressure under normal circumstances (Opp, 2005).

To investigate possible causes of oscillatory state switching within the hippocampal network we used a power spectrum-based state-space approach. By plotting each epoch according to two spectral ratios and classifying them as NREM or REM based on their position within the resulting state-space, we were able to study the relation between the states and within-state dynamics. Similar state-space approaches have previously been used to study vigilance state dynamics in humans, mice, and rats (Gervasoni et al., 2004; Diniz Behn et al., 2010; Imbach et al., 2016; Schoch et al., 2017). The distance between NREM and REM clusters was significantly reduced in septic animals. Such increased similarity between distinct oscillatory states has not been described in sepsis patients or animal models so far. Similar decreases in interstate distances have been observed in narcolepsy patients and in orexin knockout mice, expressing a narcolepsy-like phenotype that is characterized by a high level of state fragmentation (Diniz Behn et al., 2010; Schoch et al., 2017). Reduced inter-state distances in state-space could therefore be a general characteristic of high state instability, and may indicate altered attractor dynamics between stable states, facilitating the state transitions.

We found that decreased state-space distance between NREM and REM clusters was driven by opposing effects of LPS on these states within lower frequency ratio R1. In NREM, this consisted of decreased δ power and a shift toward faster oscillations within that frequency range. Decreases in NREM δ power have previously been described in sepsis in rats (Baracchi et al., 2011), although effects of lower doses of LPS on NREM δ power are less consistent (Lancel et al., 1995; Kapás et al., 1998).

Increased δ and θ power, with high interindividual variability combined with a general slowing of oscillatory activity in the REM-like state under urethane is in line with slowing of EEG activity during awake, or wake-like states, described in sepsis patients (Straver et al., 1998; Rosengarten et al., 2012; Götz et al., 2016; Urdanibia-Centelles et al., 2021). Slowing was associated with poor outcomes, including delirium (Urdanibia-Centelles et al., 2021) and death (Yamanashi et al., 2021). In animal models, increased wake δ power (Kafa et al., 2010), slowed EEG (Kurita et al., 2010), and reduced θ frequencies have been described (Mamad et al., 2018). Slowed EEG may persist in former sepsis patients (Semmler et al., 2008), but long-term effects in animal models are less consistent (Gao et al., 2017a; Ji et al., 2020b; Wang et al., 2020). In addition to changes in lower frequencies, we found no changes in γ oscillations in the REM-like state in our experiments. This is in line with results from awake animals, where hippocampal γ was unaffected by acute systemic inflammation (Mamad et al., 2018), but reduced cortical γ band power has been described at later time points (Ji et al., 2020a,b). However, caution must be taken in interpretating γ oscillations under anesthesia, as they likely do not reflect the same physiological processes underlying cognitive function as in wakefulness.

It is unclear whether these previously described power spectrum changes are because of altered background spectrum or specific oscillatory activity, as the peak frequencies within spectral power bands were typically not analyzed. The occurrence of isolated δ and θ waves in the wake EEG of septic patients suggests that they are at least in part related to oscillations (Bolton, 1987; Oddo et al., 2009; Kaplan and Rossetti, 2011). Unfortunately, most of the discussed papers limit their spectrum analysis to only one vigilance state, or in some cases to a single frequency band of interest. Where oscillatory activity is discussed, spectra were typically not decomposed into periodic and aperiodic components. Our results suggest, however, that oscillations are a main contributor to sepsis-related changes in power spectra in our model, while background shifts in E-I balance, as indicated by spectrum slope (Gao et al., 2017b) were less prominent. Further studies with both sleep and wake states are required to further elucidate NREM-REM interactions and the role of oscillatory and aperiodic brain activity in sepsis.

One hypothesized cause of increased state switching is state instability, as evidenced by high epoch-to-epoch variability within the states, resulting in high state-space jitter values. We found higher within-state velocities in the higher frequency spectral ratio (R2) after LPS injection and not in the lower frequencies where we observed the largest overall shifts in cluster location.

Changes in cortical higher frequency phenomena like spindles and HFOs signal NREM-REM transitions in normal sleep (Sánchez-López et al., 2018). Increased variability in higher EEG frequencies, even outside of the transition periods themselves, may help drive increased state switching in our animals as well. The relation between within-state jitter and sleep state stability is not entirely straightforward. Increased within-state velocities were previously observed in orexin knockout mice, but not in narcolepsy patients, despite high degrees of state fragmentation in both (Diniz Behn et al., 2010; Schoch et al., 2017). In Parkinson’s disease, which is characterized by abnormally low velocities, lower within-state velocities were associated with decreased arousability rather than changes in gross sleep architecture (Imbach et al., 2016). Even so, higher within-state velocities seem to be generally related to instability, although this does not always rise to the level of increased vigilance state changes in these differing pathologies. Additionally, we found that in our sepsis model, the state transitions themselves remained normal. State-space parameters like cluster distance, jitter and transition quality can thus each be affected differentially according to underlying pathophysiology. Further research will be needed to elucidate mechanisms underlying these changes in acute inflammatory conditions like sepsis.

We performed recordings under urethane anesthesia, which is known to largely spare the ongoing brain oscillations and to mimic a number of physiological features of natural NREM and REM sleep (Clement et al., 2008; Pagliardini et al., 2013). This offers the possibility to analyze their fine kinetics in the absence of many peripheral inputs, pain, and awakening responses that complicate the study of sleep-wake-like rhythms in ICU settings and in nonanesthetized animals. Our reduced complexity model captures many of the features of sepsis-associated changes in brain activity, but not all. Acute suppression of REM sleep is a common effect of inflammation under nonanesthetized conditions in addition to fragmentation of the remaining NREM sleep and wakefulness (Opp, 2005). Although we do not find lower amounts of the REM-like urethane state in the current study, we do find severe state fragmentation and spectral changes observed in other studies.

Memory impairments are commonly described in sepsis, but the role of disturbed sleep remains to be fully explored. Given the role of sleep in maintaining a functional immune system and its association to memory consolidation, it is crucial to better understand sleep disturbances during sepsis. Remedying sleep fragmentation and instability within hippocampal networks could be a potential treatment option for recovery of memory impairments and cognitive function in sepsis survivors. Here, we aimed to get a step closer to understanding these sleep state dynamics.

## Synthesis

Reviewing Editor: Erica Glasper, The Ohio State University Decisions are customarily a result of the Reviewing Editor and the peer reviewers coming together and discussing their recommendations until a consensus is reached. When revisions are invited, a fact-based synthesis statement explaining their decision and outlining what is needed to prepare a revision will be listed below. The following reviewer(s) agreed to reveal their identity: Daniel José Pinheiro, Jianjun Yang.

**Overview**: In this study, the authors characterize the properties and dynamics of brain oscillatory states following sepsis-associated encephalopathy (SAE) in an acute rat model of sepsis because of high dose LPS (10 mg/kg). The authors examined hippocampal oscillatory activity while rats were under urethane anesthesia, with a focus on state transition dynamics. They report increased similarity between REM-like and NREM-like states and reduced within-state stability and posit that this is a result of sepsis-related sleep fragmentation. The authors suggest that the inability to maintain stable vigilance states disrupts sleep and the accompanying restorative functions but might also have direct effects on cognition. The findings reported here are interesting, and may be of great interest to those interested in using electrophysiological data as a biomarker of neurological conditions. While the reviewers were enthusiastic about the work, the manuscript could be strengthened by addressing the concerns listed below.

**Major Concerns**

1. Results

a. Please report the survival rate of the rats following the dose of LPS used.

b. Please report the changes in body weight following administration of LPS.

c. Please add the explained variance for the principal components used in the analyses so that the reader can understand which data the component represents.

d. In addition to the lower frequency data that were reported, the authors are encouraged to also report the high frequency data.

2. Discussion

a. Please increase the discussion of the attractor dynamics. This was mentioned at the end of the abstract, but the argument is too shallow as it currently stands. The authors should add more references to this part of the discussion to help better support their hypothesis.

b. Please include a discussion of how the data presented here compare with those that have also examined the effects of repeated LPS injections on brain oscillatory states, including Ji et al., Neural network disturbance in the medial prefrontal cortex might contribute to cognitive impairments induced by neuroinflammation. *Brain Behav Immun*. 2020 Oct;89:133-144.

**Minor Concerns**

1. Overall - Please add line numbers to the document so that it is easier to denote where revisions are made.

2. Abstract - Please change “sings” in the 2nd sentence of the abstract to “signs”. A careful line edit of the manuscript should be performed to catch similar errors in word choice.
